# Bibliography

**Published:** 1893-12

**Authors:** 


### Bibliography.

A Practical Treatise on Mechanical Dentistry, by Joseph Richardson, M. D., D. D. S. Sixth Edition. Revised and edited by George W. Warren, D. D. S., with six hundred illustrations.

This edition of this invaluable work has just come to hand, too late for an extended review in this number of the Register. We shall give a more extended notice in the next number. For the present, suffice it to say that it has been much improved over former editions.

